# The effectiveness of continuity of care in patients with inflammatory bowel disease: a systematic review

**DOI:** 10.1186/s12876-023-03109-3

**Published:** 2024-01-08

**Authors:** Zijun Gu, Junyi Gu, Ping Liu

**Affiliations:** 1 Department of Otorhinolaryngology & Clinical Allergy Center, The First Affiliated Hospital, Nanjing Medical University, Nanjing, P. R. China; 2 Health School attached to Shanghai University of Medicine & Health Sciences, Shanghai, P. R. China

**Keywords:** Inflammatory bowel Disease, Continuity of care, Meta-analysis, Systematic review

## Abstract

**Aim:**

To investigate the effectiveness of continuity of care in patients with inflammatory bowel disease.

**Background:**

The prevalence of inflammatory bowel disease(IBD) is increasing by years, especially in China. Moreover, IBD is prolonged and difficult to heal, which seriously impairs the quality of life of patients. Some studies have identified that continuity of care could contribute to the improvement of the quality of life, but the results remains inconclusive in patients with IBD.

**Methods:**

PRISMA guidelines was the outline of this study. Review Manager Software (version 5.3) was used to carry out the data analysis. Outcome assessments included quality of life (QoL), remission rates, number of outpatient clinic visits, and medication adherence.

**Results:**

Ultimately, 12 studies involving 2415 patients were brought into this meta-analysis. The results indicated there was no significant difference for continuity of care to improve the QoL in intervention group (SMD = 0.02, 95% CI: -0.08, 0.12). Besides, the remission rates of disease had no difference with those patients in the two groups (OR = 1.07, 95% CI: 0.72, 1.60). However, continued care could contribute to the number of outpatient clinic visits (MD = -0.84, 95% CI: -1.19, -0.49) and patients’ adherence to medication significantly (OR = 2.40, 95% CI: 1.16, 4.95).

**Conclusions:**

IBD patients could benefited from continuity of care with reducing their number of clinic visits and improving medication adherence. Nonetheless, there was no evidence of continuity of care contribute to QoL and remission of disease for these patients.

**Supplementary Information:**

The online version contains supplementary material available at 10.1186/s12876-023-03109-3.

## Introduction

Inflammatory bowel disease (IBD), mainly comprising of ulcerative colitis (UC) and Crohn’s disease (CD) [1], is a chronic inflammatory gastrointestinal disease. IBD was considered highly associated with western habits, in that, it was called western disease [2]. It is estimated that about 1 million people in the US and 2.5 million in Europe have IBD [3]. However, with the transform of diet and living habits, a gradual increase in the incidence of IBD is more evident in Asian populations [4]. In addition, studies have shown that, currently, China has the largest population of IBD patients in Asia. Therefore, IBD has become a global disease [5]. The pathogenesis of IBD is highly diverse. It is also characterized by erratic relapses and remissions. And there is no clinical cure for IBD. IBD patients have to live with this long-term chronic disease and rebuild their lives [6]. The lifelong disease experience can greatly impair the quality of life (QoL) of those IBD patients [7]. It can effectively reflect IBD patient’s living condition, including their physical condition, psychological and social well-being and it can also be an indicator in monitoring care quality [8, 9]. There are different methods trying to improve the QoL of these patients, including continuity of care.

Continuity of care has a very broad and varied definition. Currently, the most widely accepted view is advanced by Jeannie L Haggerty. Haggerty proposes that continuity of care occurs when healthcare events are experienced by patients as coherent, connected and consistent with their complex care needs [10]. There are three dimensions in continuity of care, including informational, management and relational continuity. Informational continuity is recognized as the effective sharing and using of patients’ personal information among health service providers. Management continuity is consistent and timely coordination of care and services. Relational continuity has been defined as a long-term, ongoing therapeutic relationship of patient-provider between different healthcare episodes (11–12). Continuity of care emphasizes information and relationship transfer and coordination of patients’ care over time. It comes up with not only the exchange of patient information between different medical institutions and personnel, but also the continuity of patient care from hospital to home [13]. The advantage of this way lies in the ability to establish a continuous relationship between the nurse and the patient, and to provide the patient with personalized care to the greatest extent [14]. Continuity of care has been considered as an indispensable measure to prevent readmission and may improve QoL in patients with chronic disease [15]. Several studies have also demonstrated not only the health care providers but also patients requiring long-term care benefit from the implementation of the continuity of care conclusively [16]. Besides, studies showed that continuity of care can integrate all aspects of medical resources and reduces health cost effectively, as well as optimize patients’ awareness about their diseases and self-care behavior. Eventually, it can improve those patients’ QoL [17].

However, in the in-depth study, we found that there is a lack of unified standards for the intervention content of continuity of care for IBD patients, and there are certain differences in the evaluation of intervention effects. Continuity of care could improve the IBD patients’ clinical outcomes? It remains very controversial for this moment [18, 19]. In addition to the inconsistency of research results, there is also a lack of comprehensive evaluation on the effect of continuity of care for IBD patients. Therefore, in this study, our goal is to update and synthesize findings on continuity of care for patients with IBD and evaluate what is the most popular way of continuity of care nowadays. What’s more, we also want to investigate whether continuity of care could contribute to the IBD patients’ disease outcomes or other aspects of their disease.

## Methods

We took place a systematic review and meta-analysis to analyze the results of the randomized controlled trials. The study was carried out by acting in accordance with the Preferred Reporting Items for Systematic Reviews and Meta-Analyses (PRISMA) [20]. A protocol was previously registered on PROSPERO (CRD42021276040) [21].

### Eligibility criteria and search strategy

Two of the investigators (ZJ and JY) systematically searched relevant literature published between the inception of each online databases and August 2022 from the following databases: PubMed, Embase, the Cochrane Library, CINAHL, Web of Science and Registers Clinicaltrials. Gov, with restriction to English language. Otherwise, we went over all potentially eligible studies, including articles, reviews, meta-analyses and systematic reviews, to ensure a comprehensive search. The following medical subject heading (MeSH) terms and free text terms in various combinations were used for framing the search strategy: (“Continuity of Patient Care” OR “Long-Term Care” OR “Home Care Services” OR “Delivery of Health Care, Integrated”) AND (“Crohn’s disease” OR “colitis, ulcerative” OR “inflammatory bowel diseases” OR “CD” OR “UC” OR “IBD”). Only randomized controlled trials in human were considered to include in the analysis.

### Study selection and data collection

This review included only RCT with following inclusion criteria: Types of participants: patients (≥ 18 years) diagnosed with IBD. Studies on cancer or psychiatric patients or other chronic diseases were excluded due to the special illness situation of those patients. Types of intervention: continuity of care interventions provided by any healthcare professional during and after hospital discharge. According to the connotation of continuity of care, to be included, the interventions had to address at least one type of informational or management or relational continuity. Types of outcome: at least including one of the quality of life or remission rates or number of outpatient clinic visits or medication adherence in the results evaluation. Considering that data from various studies will be combined and analyzed, studies with clear data descriptions are selected for inclusion in this study as far as possible. Two investigators (ZJ and JY) filtrated the title and abstract of retrieved literature to exclude the irrelevant studies independently. Then they determined if the articles were eligible for inclusion by reviewing the full text. All disagreements were discussed by the two investigators (ZJ and JY). Search strategies were checked by three investigators (ZJ, JY, PL).

### Quality appraisal

All included studies were evaluated for their quality and risk of bias by two independent investigators (ZJ and JY) according to the Cochrane Risk of Bias Tool for RCTs, which contains: generation of the random sequence, allocation concealment, blinding of participants and personnel, blinding of outcome assessments, incomplete outcome data, selective reporting and other potential sources of research bias [22]. Each item needed to be judged by the investigator (JY) and given a conclusion of high, low or unclear risk of bias. Besides, the quality of the evidence would be rated as grade A, B, and C. Studies with grade C were excluded for further analysis. Any discrepancies were resolved by discussion.

### Data extraction

One investigator (ZJ) performed the data extraction. First-author, publication year, country, type of disease, sample sizes, mean age, duration of follow-up, intervention methods, outcome were extracted into a form. The second investigator (JY) checked the data for completeness and accuracy. If no data which could be analyzed directly was reported, the raw data of the study was used to calculate the eligible data.

### Synthesis

A meta-analysis, aimed to estimate the efficacy of continuing care using Review Manager Software (version 5.3), was carried out based on the extracted data of two or more studies. For dichotomous data, using Mantel-Haenszel (MH) chi-square test to calculate the odds risk (OR) and its 95% confidence interval (CI). Inverse variance (IV) statistical method was used for continuous data. Statistical data were summarized using mean difference (MD) and standardized mean difference (SMD), depending on the consistency of the measures used in the study results [23]. Heterogeneity between studies was assessed by I^2^ statistics and the Chi-square test. If I^2^ value was ≥ 50% or *P* was < 0.10, we can define it has a significant heterogeneity. A random-effects model was employed in research of significant heterogeneity, otherwise we undertook a fixed-effects model to synthesize the data [24]. To explore the potential bias sources, sensitivity analysis and subgroup analysis were carried out. Forest plots were generated to display pooled estimates. Publication bias was not investigated due to each outcome including less than 10 studies [25].

## Results

### Search results

A total of 5082 articles and 448 registers were yielded by searching the electronic database. After removing the duplicates, 3780 records were screened by title and abstract. After excluding all of the irrelevant records, 156 studies were left for full manuscripts review. Of those records, 145 were excluded because they did not meet the pre-set criteria. At least, 11 articles were covered in our meta-analysis [26–36]. On account of Elkjaer et al. reported two separate RCTs carried out in Denmark and Ireland in an article, we analyzed the data as two independent studies. The study selection is summarized in Fig. S1.

### Methodological quality assessment of studies included

Methodological quality of all the 12 studies meeting our inclusion criteria were assessed as grade B. The outcomes were shown in Table 1. Two studies did not include the process of randomization sequence generation. Allocation concealment was judged high risk of bias for one study which illustrated didn’t conduct allocation concealment clearly, and four studies were ranged in unclear risk of bias. High risk of bias was found mainly in the deficiency of blinding. Only three studies mentioned using blinding, two of which reported the participants and study personnel were blinded while the remaining one were reported the outcome assessors were blinded. The rest of the articles were rated as high or unclear risk of performance and detection bias. Two studies were ranged in having high risk of bias for incomplete outcome data. Besides, in data analysis, they did not employ intention-to-treat principles to ensure the scientificity of the study and provide sufficient information to follow up. There were no identified selective reports and other potential biases in any of the included studies. The Fig. S2 summarized the risk of bias assessment for the included studies.

**Table 1:** Study characteristics

Authors (year)	Quality of studies included	Country	IBD type	Study design	Participants (Intervention group/Control group)	Age (Intervention/Control)	Duration of follow-up (months)	Intervention	Control	Main outcomes (Measurement tools)
Andrew McCombie et al. (2020) [26]	B	New Zealand	UC + CD	Parallel-RCT	50/50	35.2 ± 12.4/34.3 ± 12.9	12	smartphone app (IBDsmart and IBDoc)	standard care	QoL (IBDQ), number of outpatient clinic visits
Raymond K. Cross et al. (2018) [27]	B	USA	UC + CD	Parallel-RCT	231/117	40.1 ± 11.7/38.2 ± 12.5	12	Web-based tele-management system (TELE-IBD System)	standard care	QoL remission rate, number of outpatient clinic visits
Raymond K. Cross et al. (2012) [28]	B	USA	UC	Parallel-RCT	25/22	40.3 ± 14.4/41.7 ± 13.9	12	Home tele-management (UC HAT)	best available care	QoL (IBDQ), remission rate, medication adherence
Margarita Elkjaer et al. (2010) [29]	B	Ireland	UC	Parallel-RCT	52/48	42 (18–68)/48 (19–95)	12	Web-based tele-management system (http://www.constant-care.dk.)	conventional treat-ment and follow-up in the IBD out-patient clinic	QoL (SIBDQ), medication adherence
Margarita Elkjaer et al. (2010) [29]	B	Denmark	UC	Parallel-RCT	117/116	41 (21–69)/48 (21–69)	12	Web-based tele-management system (http://www.constant-care.dk.)	conventional treat-ment and follow-up in the IBD out-patient clinic	QoL (SIBDQ), medication adherence
Alan C. Moss et al. (2009) [30]	B	USA	UC	Parallel-RCT	21/60	44 ± 16/47 ± 17	6	Patient Support Program	standard care	QoL (SIBDQ), medication adherence
Anna-Maija Puolanne et al. (2019) [31]	B	Finland	UC + CD	Parallel-RCT	63/60	NA	12	home monitoring	control care	QoL (15D-questionnaire), number of outpatient clinic visits
Guillaume Bonnaudet et al. (2017) [32]	B	France	UC + CD	Parallel-RCT	29/25	32.7 ± 10.9/32.7 ± 12.6	12	Web-based tele-management system and smartphone app (EasyMICI–MaMICI)	standard care	QoL (SIBDQ), remission rate, number of outpatient clinic visits
Marin J de Jong et al. (2017) [33]	B	Netherlands	UC + CD	Parallel-RCT	465/444	44.0 ± 14.1/44.1 ± 14.2	12	Web-based tele-management system (myIBDcoach)	standard care	QoL (SIBDQ), number of outpatient visits, medication adherence
Matthew Schliep et al. (2020) [34]	B	USA	UC + CD	Parallel-RCT	145/72	39.5 ± 12.0/38.3 ± 12.5	12	Web-based tele-management system (TELE-IBD)	standard care	QoL (SF-12)
Javier Del Hoyo et al. (2018) [35]	B	Spain	UC + CD	Parallel-RCT	42/21	39.31 (22–61)/41.12 (19–66)	6	Web-based tele-management system (TECCU)/Nursing care by telephone	usual care	QoL (IBDQ-9), remission rate, number of outpatient clinic visits, medication adherence
Lars-Petter Jelsness-Jørgensen et al. (2012) [36]	B	Norway	UC + CD	Parallel-RCT	71/69	43.0 ± 16.1/46.2 ± 15.5	12	Nurse-led follow-up	Conventional follow-up	QoL (IBDQ)

IBDQ, Inflammatory Bowel Disease Questionnaire; SIBDQ, Short Inflammatory Bowel Disease Questionnaire; SF-12, 12-item Short Form Health Survey; IBDQ − 9, Inflammatory Bowel Disease Questionnaire; NA, not applicable

### Main characteristics of the eligible studies

Table 1 showed main features of the 12 parallel-RCTs comprising of 2415 patients. The publication data ranged from 2009 to 2020. In these studies, the age of the patients participating ranged from 32.7 to 48 years old. The shortest follow-up was 6 months, whereas the longest follow-up lasted 12 months. Nine studies compared distance management with standard care, while another three trails compared patient support program, home monitoring and nurse-led follow-up with standard care, control care, and conventional follow-up, respectively. The majority of trials did not describe specific details, such as the timing of follow-up or the method of patient education in the control group. Cross et al. and Schliep et al. undergone three arms trials which control group was standard care and intervention group receive telemedicine education in different frequency. At the same time, Hoyo et al. trial were three arms too which compared the efficacy of web-based tele-management system, telephone nursing care, and usual care. We merged intervention group data in the three trials for meta-analysis, because we did not pay close attention to the frequency and form of continuity of care.

### Effect of the intervention

#### Primary outcome: quality of life

**Fig. 1:**
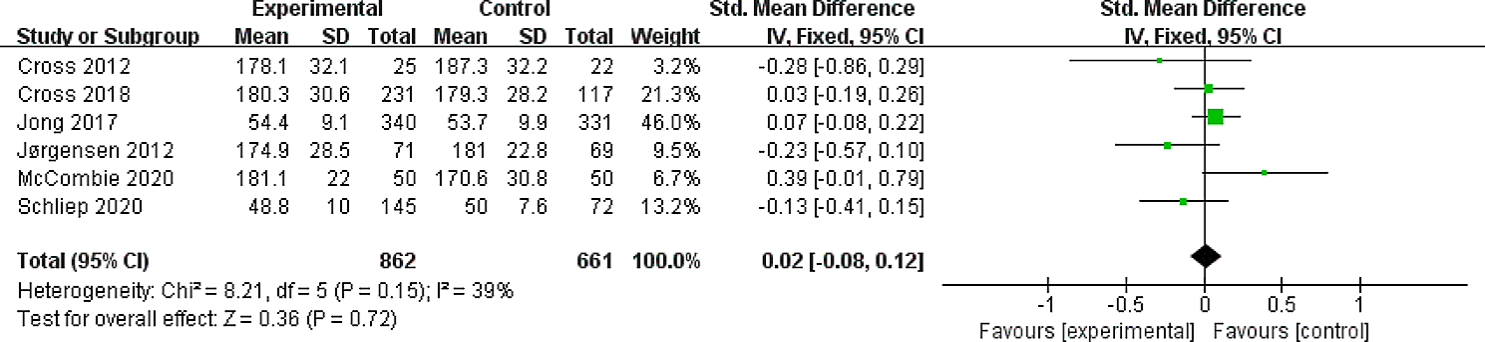
Forest plot of the fixed-effects meta-analysis evaluating the effect of continuity of care interventions on QoLCI: confidence interval; IV: Inverse variance

All studies took quality of life as primary or secondary outcomes, of which 5 of the studies had data gaps that ultimately prevented them from being combined and 1 trail calculated the scores in error. As a result, a total of 6 studies’ data were included for analyzing in the meta-analysis for quality of life [26–28, 33, 34, 36]. Four trials reported improvements in IBDQ scores one year later [26–28, 36], another 2 trails used SIBDQ [33] and SF-12 [34] to report the level of quality of life after one year, respectively. A fixed-effects meta-analysis of the studies showed that continuity of care did not significantly improve QoL (SMD = 0.02, 95% CI: -0.08, 0.12), with no certain heterogeneity (I^2^ = 39%, *P* = 0.15) (Fig. 1). In addition, we deleted every eligible study one by one to carry out sensitivity analysis. The results were similar and the combined results were highly reliable.

#### Secondary outcome: remission rates

**Fig. 2:**
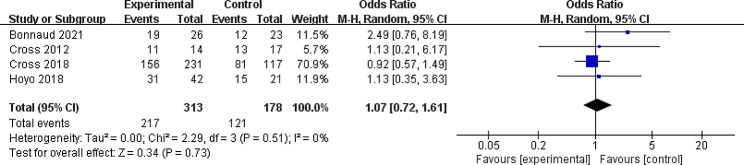
Forest plot of the fixed-effects meta-analysis evaluating the effect of continuity of care interventions on remission ratesCI: confidence interval; M-H: Mantel-Haenszel

Four studies provided data on the rates of remission in 491 IBD patients; a forest plot of their results is shown in Fig. 2, which illustrated no statistical effect on this outcome was found from combining these studies (OR = 1.07, 95% CI: 0.72, 1.60) with no heterogeneity (I^2^ = 0%, *P* = 0.51) [27, 28, 32, 35]. The results remained stable after sensitivity analysis. It demonstrated the robustness of pooled data of the review process.

#### Secondary outcome: number of outpatient clinic visits/patient/year

**Fig. 3:**
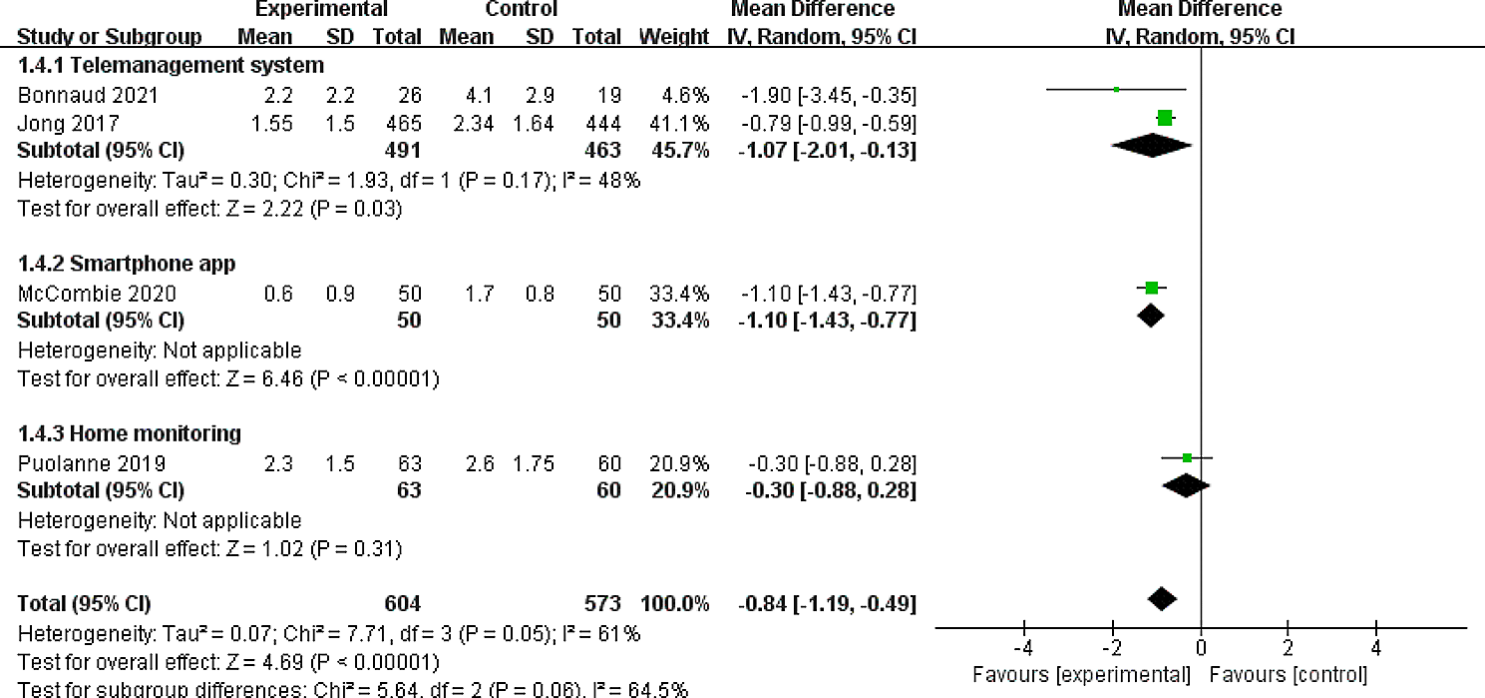
Forest plot of the Random-effects meta-analysis evaluating the effect of continuity of care interventions on number of outpatient clinic visitsCI: confidence interval; IV: Inverse variance

Six studies reported the number of outpatient clinic visits in IBD patients after continuity of care intervention (26–27, 31–33, 35). However, 2 trials did not perform sufficient information to allow for meta-analysis, so they were not included in the analysis [27, 35]. The rest of the four articles offered appropriate data [26, 31–33]. According to the results of random-effects meta-analysis, a significant reduction was observed in the visits of outpatient clinic in continuity of care group compared with standard care (MD =-0.84, 95% CI: -1.19, -0.49) during one year. Effect sizes varied, and the I^2^ of 61% suggested the presence of heterogeneity (I^2^ = 61%, *P* = 0.05). A sensitivity analysis found that dropping any single studies was not associated with decreased heterogeneity. When subgroup analysis was assessed by type of intervention, the heterogeneity decreased a little (I^2^ = 48%, *P* = 0.17) that indicated type of intervention were a source of heterogeneity probably. The subgroup analysis also suggested significant differences in the intervention of tele-management system and smartphone app (MD =-1.07, 95% CI: -2.01, -0.13, and MD =-1.10, 95% CI: -1.43, -0.77, respectively, Fig. 3), whereas there was no certain difference between groups in home monitoring (MD =-0.30, 95% CI: -0.88, 0.28).

#### Secondary outcome: medication adherence

**Fig. 4:**
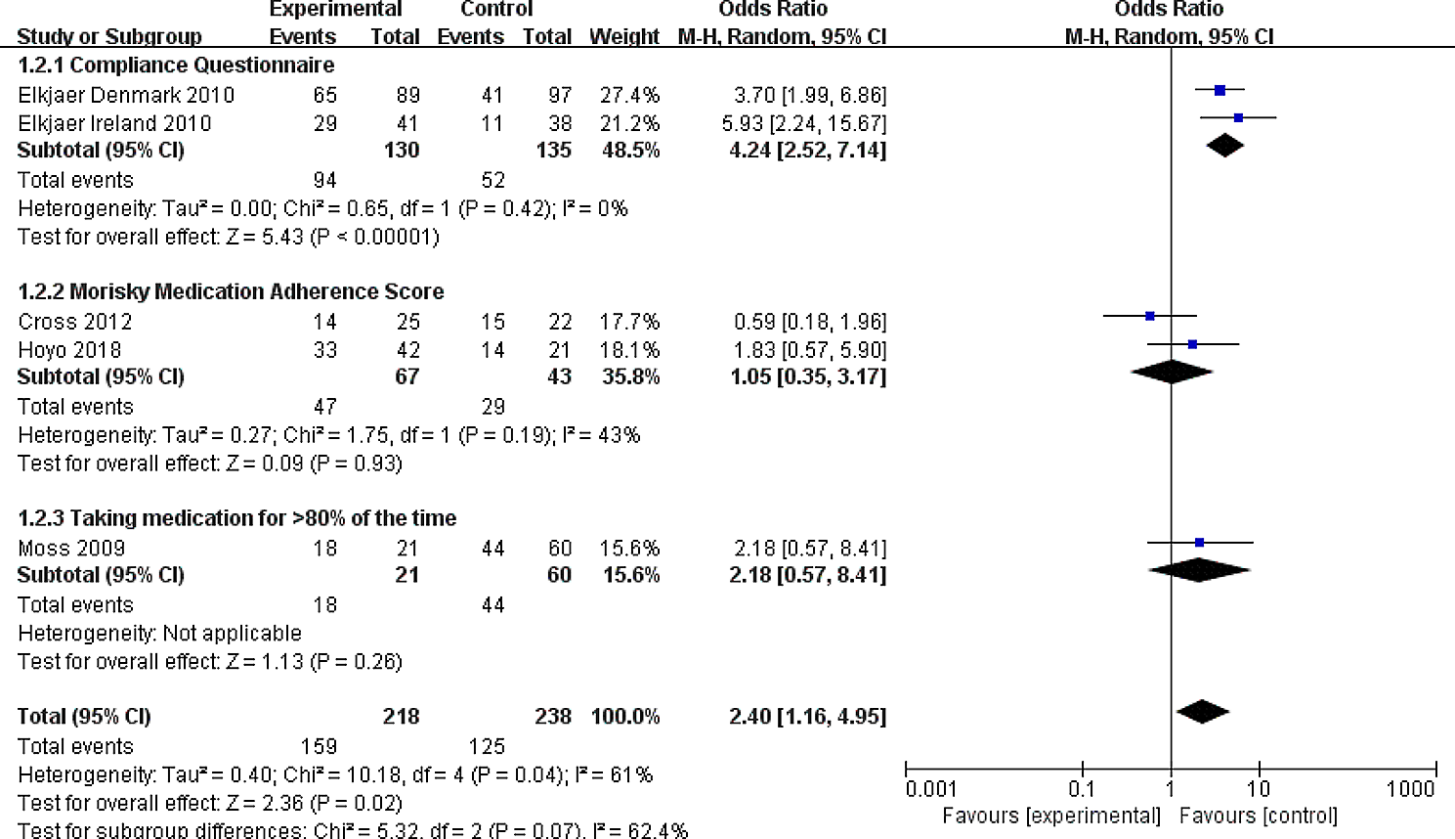
Forest plot of the Random-effects meta-analysis evaluating the effect of continuity of care interventions on medication adherenceCI: confidence interval; M-H: Mantel-Haenszel

**Fig. 5:**
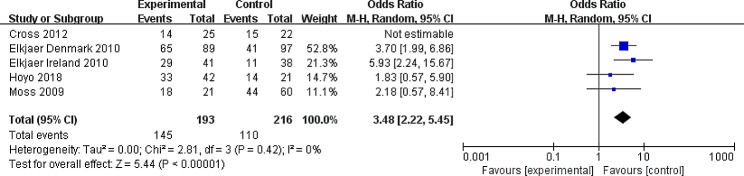
Forest plot of the sensitivity analysis excluding Cross et al. study published in 2012CI: confidence interval; M-H: Mantel-Haenszel

Six RTCs reported on the medication adherence [28–30, 33, 35]. Due to the certain differences in the way data are reported, Jong et al’s data could not be pooled with the others [33]. They reported the target outcome with continuous data, while other authors used dichotomous data. Finally, five studies were included in the meta-analysis [28–30, 35]. Compared to standard care, continuity of care could improve the adherence of taking medication (OR = 2.40, 95% CI: 1.16, 4.95), however, there was heterogeneity (I^2^ = 62%, *P* = 0.04). Based on instrument of adherence measurement, the results of the subgroup analysis reducing the heterogeneity indicated that different measure instrument could be the source of heterogeneity. Furthermore, the heterogeneity decreased significantly (I^2^ = 0%, *P* = 0.42) by excluding Cross et al’s study published in 2012, and this analysis suggested effects of continuity of care (OR = 3.84, 95% CI: 2.22, 5.45) with an enhanced effect size (Figs. 4 and 5).

## Discussion

This meta-analysis was conducted to examine the effectiveness of continuity of care in patients with IBD. At the beginning of the study, we proceeded a detailed literature selection and quality evaluation. The included articles were evaluated with the Cochrane risk bias assessment tool and 12 studies were rated as grade B. There are various ways of continuity of care such as nurse-led follow up, telephone-follow up, telemedicine and mixed intervention [37]. However, in this research, it suggests that telemedicine has become the most popular continuous care way in recent years [38]. Almost two thirds of the included literatures used telemedicine to make changes to the IBD patients about their QoL or the other aspects of their disease.

Previous studies have proved that continuity of care could contribute to their medical satisfaction [39], health promotion behavior [40], adherence to medication [41] and hospital use [42]. However, in our study, the results indicated that continuous care did not effectively improve the QoL (SMD = 0.02, 95% CI: -0.08, 0.12) of patients with IBD. This result remains relatively consistent after heterogeneity test carried out in these studies. Therefore, our results suggest continuous care cannot significantly improve the QoL of IBD patients. Nevertheless, there is still the possibility of bias in results caused by intervention methods and basic information of the patients. Moreover, continuity of care did not show a significant effect in improving or reducing the remission rates in these patients as well. Meanwhile, the result demonstrated continuity of care by telemedicine platform was a comparatively secure care model which didn’t lead to aggravation of disease symptoms [43]. The pooled data has proved it robustness during the sensitivity analysis. In addition, only 4 of the included studies provided relevant data on remission rate for analysis. As a result, the amount of data available for analysis is small, which should have more studies to clarify the effectiveness of continuous care in the remission rates of their disease.

When we synthesized the results of other studies, our study found that continuity of care could decrease the patients’ hospital visits (MD =-0.84, 95% CI: -1.19, -0.49). In addition, it can improve the patients’ level of medical compliance effectively [44, 45]. However, the data of the studies had great heterogeneity (I^2^ = 61%, *P* = 0.05) due to the homogenization and transformation of the data, with the merger. After analyzing the sources of heterogeneity in these literatures, we concluded that the intervention method was the reason for the huge difference. Then, we conducted another subgroup analysis, and the results of it showed the intervention of continuous care was still very positive and meaningful, as well as the stable results. Patients using mobile applications and web-based platforms showed a large gap in this study, which may be due to differences in the content and form of telemedicine. Compared with the web-based platform, applications had higher convenience [46]. It is not constrained by time and place, which can be more expedient for patients to use [47]. Nevertheless, no difference in medical compliance was observed in the home-monitoring care group compared with web-based platforms (MD =-0.30, 95% CI: -0.88, 0.28). Exploring the reasons may be under these two methods, the number of outpatient visits or the frequency of medical resource use of IBD patients did not improve significantly. There is no difference in convenience between these two intervention methods, and compared with telemedicine, these two intervention methods do not have their advantages as well. In terms of medical compliance, our study also came to very positive conclusions. We believe that continuous care can improve the treatment compliance of patients with IBD [48]. Although there is some heterogeneity in the data, we also found a similar source of heterogeneity through analysis-intervention methods. In view of the heterogeneity of data, sensitivity analysis of data elimination was also conducted for every single literature. When Cross’s study conducted 2012 was removed, the results reached stability. After in-depth analysis of this article, we found that the heterogeneity of this study may be its small sample size, with only 14 patients in the intervention group receiving final data collection, while 21 patients in the control group.

Nowadays, telemedicine has become the most popular way to carry on the continuous care for those patients with chronic diseases [49]. Compared to other means of intervention, telemedicine has its unique advantages [50, 51]. Application (APP) is a common carrier of telemedicine, it has provided IBD patients with a useful adjunct to their disease management [52]. It created not only a lot of convenience for those patients who live a long distance from their health provider but also reduced other costs and risks incurred by travelling for medical treatment [53]. It can serve people’s health requirement anytime, as long as you have a phone or a tablet. It is not limited to web pages and can be used even without the internet. Especially in the context of COVID-19, it is even more indispensable for people with IBD to stay at home and away from crowd to prevent infection and stay healthy [54]. In the meantime, safety of patients remains a concern with continuous care by means of telemedicine platform management. Safety issues mainly involves poor design of information management platform. Encouraging medical staff to report problems associated with the telemedicine platform, engaging patients in their own care and utilizing electronic medical record or personal health record what is an interactive tool are maybe some effective solutions to improve patient safety [55, 56].

Now, there have also been research claimed that telemedicine cannot contribute to the IBD patients’ quality of life [57]. Even those patients identified benefits for the telemedicine, including a greater knowledge o of the disease. It can also help them with their symptoms monitoring. Moreover, patients can strengthen the connection with their health care provider and increases their satisfaction with medical care as well. Some researchers are looking into the reasons why patients’ QoL have no relationship with the telemedicine [58]. It claimed the reason may be that the patient’s continuous care compliance has decreased during this time or the saturation of continuous care information within a few months. From a software design perspective, some scholars believe we need to provide those patients with more diverse and personalized systems. Designing such a tele medical method may be more attractive to such patients, and more able to help them manage their disease and improve their QoL [59].

Patients with IBD often experience complex disease changes, and because of the nature of the disease, they need to carry out a life-long disease management [60]. In this case, the existence of continuity of care is becoming more and more important. If it is not available, it may create a huge gap in the communication and management and affect the prognosis and rehabilitation of patients [61]. However, it is still very imperative to have more and more higher-quality studies to figure out whether continuity of care could make improvements on the IBD patients with their disease management.

### Limitations

There are a number of inevitable limitations in our article. First of all, continuity of care comprised lots of multicomponent interventions, we didn’t find out the full range of interventions for continuing care, which leaded to although as comprehensive information retrieval as possible was carried out, we might still miss potential target literature. Secondly, a few eligible studies that reported incomplete data were eliminated in our analysis, which might bring about inaccuracies to our outcomes. Finally, significant statistical heterogeneity was observed in the analysis of continuity of care effects on number of outpatient clinic visits and medication adherence. Though our subgroup analysis and sensitivity analysis found the possible sources of heterogeneity by single article, we couldn’t conduct meta-regression analysis to verify factors that may generate heterogeneity due to the limitations on the number of studies.

## Conclusion and relevance to clinical practice

In conclusion, continuity of care could not significantly improve the QoL and disease activity of patients with IBD. Whereas, it can significantly reduce the number of hospital visits of patients, improve their adherence to disease treatment and ensure the safety of IBD patients. Although there are various continuing care methods (such as nurse-led follow-up, mobile APP, patient support program, Web-based tele-management system), telemedicine has become the most popular and common way to carry out the continuing care nowadays from the perspective of frequency and effect. In the future, more personalized and intelligent remote management platforms need to be developed to promote IBD patients to maintain remission. Further validation of the positive effects of continuing care on other aspects of IBD patients and the results of this study are still needed. By compiling the existing research results, this meta-analysis might provide clinical practice with evidence of a consistent effect and develop a useful clinical guideline in the end.

### Electronic supplementary material

Below is the link to the electronic supplementary material.

Supplementary Material 1

## Data Availability

The datasets and materials analyzed during the current study are available from the corresponding author upon reasonable request.
