# Supplementary material for: The effectiveness of continuity of care in patients with inflammatory bowel disease: a systematic review
Source: BMC Gastroenterol. 2024 Jan 8;24:24. doi: 10.1186/s12876-023-03109-3 (PMC10773097; doi:10.1186/s12876-023-03109-3)
Supplement: Supplementary file 1 — Supplementary Material 1 [file 12876_2023_3109_MOESM1_ESM.docx]

**Supplementary file**

**Figure S1. Flowchart of study selection process.**

RCT, randomized controlled trial.


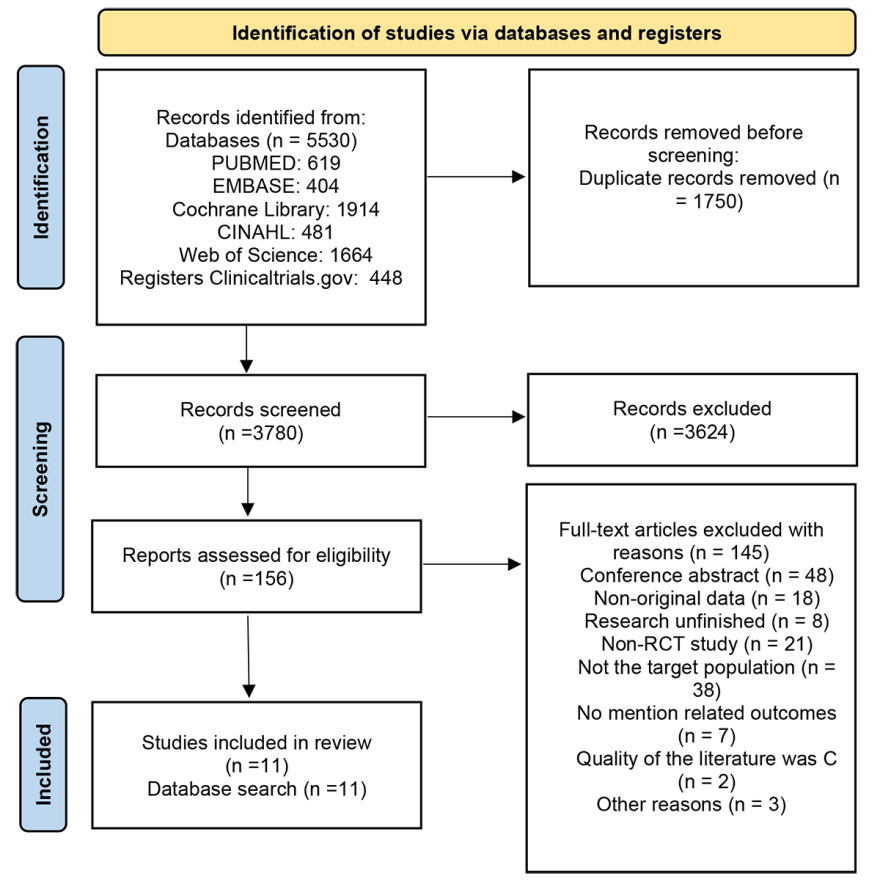


**Figure S2. Risk of bias summary: review authors' judgements about each risk of bias item for each included study.**

**
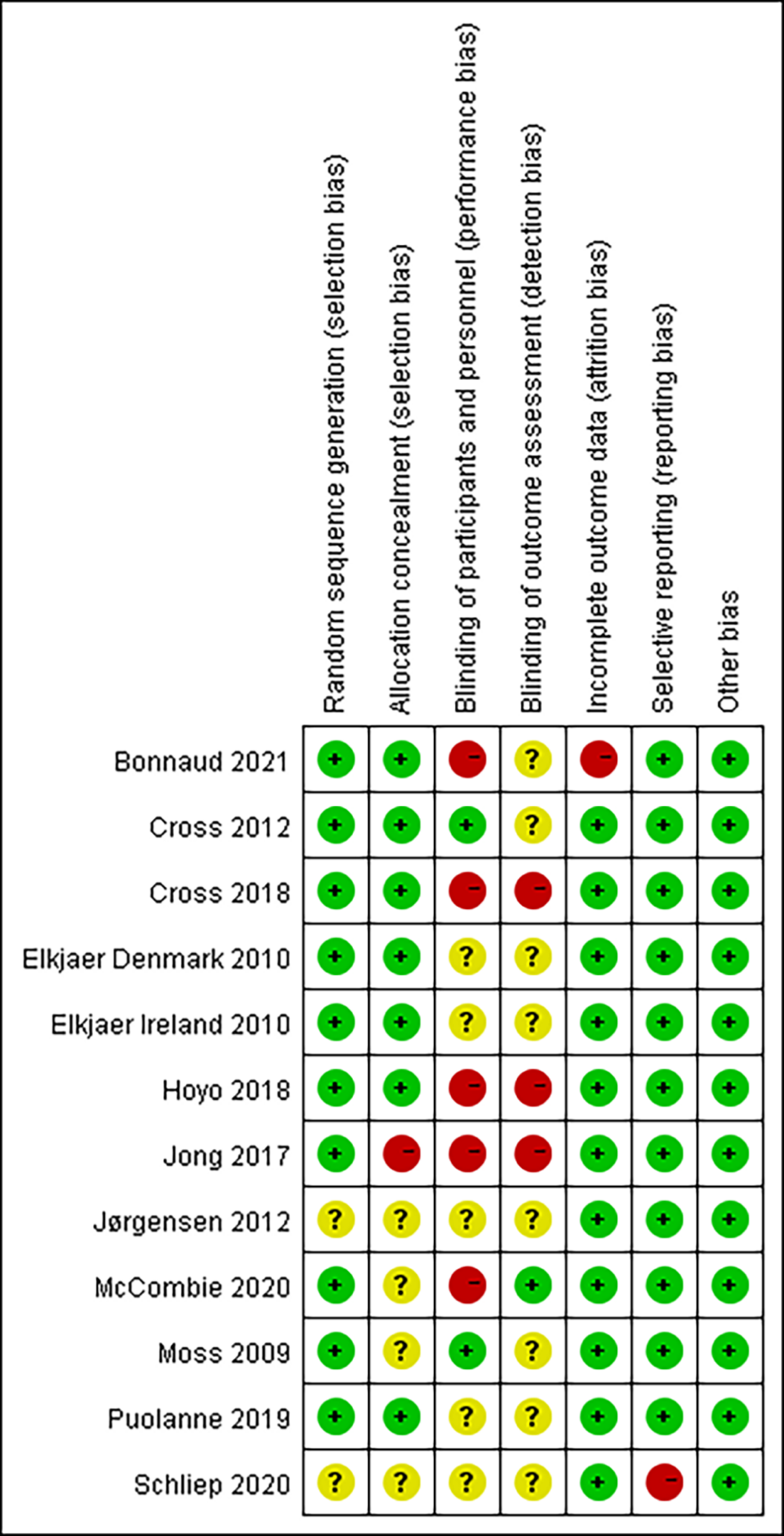
**
